# Identification of Hybrids in *Potamogeton*: Incongruence between Plastid and ITS Regions Solved by a Novel Barcoding Marker *PHYB*

**DOI:** 10.1371/journal.pone.0166177

**Published:** 2016-11-17

**Authors:** Tao Yang, Tian-lei Zhang, You-hao Guo, Xing Liu

**Affiliations:** Laboratory of Plant Systematics and Evolutionary Biology, College of Life Science, Wuhan University, Wuhan, Hubei, China; National Cheng Kung University, TAIWAN

## Abstract

*Potamogeton* is one of the most difficult groups to clarify in aquatic plants, which has an extensive range of interspecific morphological and ecological diversity. Internal transcribed spacer (ITS) is prevalent for phylogenetic analysis in plants. However, most researches demonstrate that ITS has a high percentage of homoplasy in phylogenetic datasets. In this study, eighteen materials were collected in *Potamogeton* from China and incongruence was shown between the *rbcL* and ITS phylogenies. To solve the discrepancy, we employed a novel barcode *PHYB* to improve resolution and accuracy of the phylogenetic relationships. The *PHYB* phylogeny successfully resolved the incongruence between the *rbcL* and ITS phylogenies. In addition, six hybrids were confirmed using *PHYB*, including *P*. *compressus* × *P*. *pusillus*, *P*. *octandrus* × *P*. *oxyphyllus*, *P*. *gramineus* × *P*. *lucens*, *P*. *distinctus* × *P*. *natans*, *P*. *distinctus* × *P*. *wrightii*, and *S*. *pectinata* × *S*. *amblyophylla*. Whereas, only one hybrid was identified (*P*. *compressus* × *P*. *pusillus*) by ITS, indicating that ITS homoplasy was present in *Potamogeton* and ITS was completely homogenized to one parental lineage. Thus, ITS might have limited utility for phylogenetic relationships in *Potamogeton*. It is recommended that a three-locus combination of chloroplast DNA gene, ITS and *PHYB* is potential to effectively reveal more robust phylogenetic relationships and species identification.

## Introduction

*Potamogeton* is a cosmopolitan group of aquatic herbs with submersed or floating leaves. Traditionally, species in this genus can been divided into two subgenera *Potamogeton* and *Coleogeton* [1, 2]. However, substantial researches suggest that subgenus *Coleogeton* should be elevated to the generic level and named it *Stuckenia* [3, 4]. Moreover, two morphological characteristics have been recognized: linear-leaved and broad-leaved groups [1, 5]. On the other hand, the *Potamogeton* species are also classified into heterophyllous and homophyllous types.

*Potamogeton* (including *Stuckenia*) is a typically notorious group in taxonomy due to a wide range of morphological and ecological diversity [6–8]. Furthermore, interspecific hybridization is frequent in this genus, owing to coexisting in the same ecological niche [9]. There are 69 species and more than 50 hybrids worldwide in *Potamogeton*, and there are about 28 *Potamogeton* species distributed in China [6, 10]. But only a few hybrids have been identified in this genus in China [4, 5, 11, 12]. Given several morphological characteristics for the hybrids are similar to their parents, it is always not conclusive to identify the hybrids solely based on morphology [13, 14]. Furthermore, chromosomes for the *Potamogeton* species are so small that it is difficult to apply accurate cytological assays to identify the species, including chromosome numbers counting and in-situ hybridization [4, 9, 15].

Fortunately, barcoding markers have been successfully carried out to resolve substantial mysteries for phylogenetic relationships. Internal transcribed spacer (ITS) is prominent for phylogenetic analysis in plants [16]. ITS has an advantage for phylogenetic reconstruction and species identification, including universality, simplicity, intragenomic uniformity, intergenomic variability, and low functional constraint [16]. Thus, ITS is a dominate maker in phylogenetic analysis, containing more than one third phylogenetic researches [16]. In addition, ITS is considered to be the best performing DNA barcode in *Potamogeton* compared with *rbcL*, *matK*, and *trnH–psbA* [12, 17]. Du et al. confirmed six putative hybrids using a combination of ITS and *rbcL* markers [11, 12]. Wang et al. dissected phylogenetic relationships and hybrid origin of *Potamogeton* species in China using ITS [4]. However, ITS exhibits a high percentage of homoplasy, owning to compensatory base change, paralogy, pseudogene, sequencing error, alignment problem, incomplete concerted evolution, and a combination of these phenomena [16]. Therefore, homoplasy markedly reduces its reliability for phylogenetic reconstruction and species identification.

An alternative routine is to utilize low-copy nuclear genes to improve resolution and accuracy for phylogenetic analysis [18, 19]. Low-copy genes are biparentally inherited and have lower homoplasy than ITS [16, 20]. Moreover, these genes contain codons to facilitate homologous alignments and limit alignment ambiguity. Substantial nuclear genes have been developed for phylogenetic analysis, such as argenine decarboxylase coding sequence [20], phytochrome [21–23], alcohol dehydrogenase [24, 25], *LEAFY* [26], and *CHS* [27]. *PHYB* is a group of phytochrome family and it has been used for phylogenetic constructions within Poaceae, Celastraceae and so on [22, 28]. In the present study, we selected eighteen materials in *Potamogeton* from China. We initially used a combination of chloroplast DNA (cpDNA) gene *rbcL* and nuclear ribosomal DNA (nrDNA) region ITS to investigate whether incongruence was occurred between the *rbcL* and ITS phylogenies. Then a nuclear gene *PHYB* was employed to effectively explore more robust phylogenetic relationships. This study might lay a foundation for future studies in *Potamogeton*.

## Materials and Methods

### Plant materials and DNA extraction

Initially, we confirmed eighteen materials in *Potamogeton* based on taxonomy described by Wiegleb and Kaplan [6]. Geographic coordinates of all collected samples are list in Table 1. In total, ten species were identified solely according to the taxon, including eight species in *Potamogeton* and two species in *Stuckenia* (Table 1). None of the collected samples are endangered in China and no permits are required. In addition, six putative materials were also collected in this study and we named them according to the ITS phylogenic tree except that the identified hybrids were based on parental lineage. Voucher species were deposited in the Wuhan University herbarium. We also downloaded substantial sequences from GenBank to elevate the discrimination power (S1 Table). *Ruppia maritime* was used as an outgroup in the phylogenetic analysis, which is close relative to *Potamogeton* [29].

**Table 1:** Information for the collected species of *Potamogeton* in China.

Taxon	Locality	ID number	Accession number		
			ITS	*rbcL*	*PHYB*
***P*. *compressus***	ErYuan, Yunnan; 99°57′N, 26°8′E	*P*. *compressus1*	KX062100 KX062101	KX024600	KX359814 KX359815
***P*. *compressus***	Shangguan, Yunnan; 100°5′N, 25°56′E	*P*. *compressus2*	KX062102 KX062103	KX024601	KX059477 KX059478
***P*. *oxyphyllus***	Tengchong, Yunnan; 98°34′N″, 25°12′E	*P*. *oxyphyllus1*	KX062094 KX062095	KX024597	KX359812 KX359813
***P*. *oxyphyllus***	Tengchong, Yunnan; 98°33′N″, 25°7′E	*P*. *oxyphyllus2*	KX062096 KX062097	KX024598	KX059504 KX059505
***P*. *pusillus***	Lasa, Xizang; 91°4′N, 29°39′E	*P*. *pusillus1*	KX062098 KX062099	KX024599	KX059513 KX059514
***P*. *octandrus***	Baoshan, Yunnan; 99°12′N, 25°14′E	*P*. *octandrus1*	KX062092 KX062093	KX024596	KX059501 KX059502
***P*. *gramineus***	Hongyuan, Sichuan; 102°21′N, 32°26′E	*P*. *gramineus1*	KX062104 KX062105	KX024602	KX059487 KX059488
***P*. *gramineus***	Luhuo, Sichuan; 100°13′N, 31°37′E	*P*. *gramineus2*	KX062106 KX062107	KX024603	KX059488 KX059489
***P*. *lucens***	Heqing, Yunnan; 100°10.58′N, 26°37.06′E	*P*. *lucens1*	KX062124 KX062125	KX024604	KX359810 KX359811
***P*. *lucens***	Dali, Yunnan; 100°11′N, 25°43′E	*P*. *lucens2*	KX062126 KX062127	KX024605	KX059490 KX059491
***P*. *distinctus***	Chayu, Xizang; 97°21′N, 28°37′E	*P*. *distinctus1*	KX062114 KX062115	KX024609	KX059484 KX059485
***P*. *distinctus***	Tengchong, Yunnan; 98°40′N, 25°37′E	*P*. *distinctus2*	KX062116 KX062117	KX024610	KX059485 KX059486
***P*. *wrightii***	Xiangyun, Yunnan; 100°36′N, 25°26′E	*P*. *wrightii1*	KX062112 KX062113	KX024607	KX359818 KX359819
***P*. *wrightii***	Chengjiang,Yunnan; 102°54′N, 24°37′E	*P*. *wrightii2*	KX062110 KX062111	KX024608	KX059516 KX059517
***P*. *natans***	Lasa,Xizang; 91°4′N, 29°39′E	*P*. *natans1*	KX062108 KX062109	KX024606	KX359816 KX359817
***S*. *pectinata***	Yuanmou, Yunan; 101°49′N, 25°36′E	*S*. *pectinata1*	KX062120 KX062121	KX024611	KX359806 KX359807
***S*. *pectinata***	Chuxiong, Yunnan; 101°30′N, 25°5′E	*S*. *pectinata2*	KX062118 KX062119	KX024612	KX059506 KX059507
***S*. *amblyophylla***	Xiangcheng, Sichuan; 99°32′N, 29°5′E	*S*. *amblyophylla1*	KX062122 KX062123	KX024613	KX359808 KX359809

Species nomenclature are based on Wiegleb and Kaplan (1998), and ID numbers for the selected species are named based on the ITS phylogeny.

All sampled materials were collected from fresh preserved leaf tissue and stored with silica gel. Total DNA from the dried materials (40 mg) was isolated using the Plant Genomic DNA Kit (TianGen, Beijing, China) according to the manufacturer’s instruction.

### PCR amplification and sequencing

The cpDNA gene *rbcL* was amplified using the primer 1375 and primer 26 described by Iida et al. [30]. The nuclear region ITS was amplified using ITS1 and ITS4 [31, 32]. Initially, the primers B-up and B-down were used to amplify *PHYB* according to Mathews et al. [22]. Then specific primers were designed to amplify partial *PHYB* sequences (PF: ATGTGACACAGTTGTGGACCA; PR: CATCATCCTTGTCTTCAGGGT). The PCR reaction mixtures were 50 μL, containing 10–35 ng total DNA, 2.5 mM dNTP, 1.5 mM MgCl_2_, 50 mM KCl, 5 μM forward and reverse primers, and 2 units ExTaq DNA polymerase (Takara, Dalian, China). PCR amplification profiles consisted of 5 min at 94°C for initial denaturation; 35 cycles of 1 min at 94°C, annealing for 1 min at 55°C, 1 min at 72°C for extension; and 10 min at 72°C for a final extension step. All PCR products were further purified by the High Pure PCR Product Purification Kit (Roche) according to the manufacturer’s recommendation. The purified PCR products for *rbcL* were directly sequenced in both directions using the specific primers. For the two nuclear barcodes, the purified PCR products were cloned into the PMD19-T vector following the manufacturer’s instruction (Takara, Dalian, China). Then five positive clones were sequenced in both directions. All sequencings were performed on an ABI 3730 DNA Sequencer using BigDye Terminator version 3.1 (Applied Biosystems). For identical sequences from the cloned PCR products, only one sequence was contained in the dataset. All obtained sequences in this study were deposited in GenBank (Table 1).

### Sequence analysis

Two complementary sequences were assembled using ContigExpress in Vector NTI Suite 2.0 v5.5.1 [33]. All assembled sequences were further aligned using Clustal X v2.0 [34]. Then the aligned sequences were used to construct the phylogenetic trees using maximum-parsimony (MP) and neighbor-joining (NJ) methods. MP analysis was performed using the program PAUP* v. 4.0b10 [35]. Heuristic search strategy was conducted using 1000 replicates with random taxon-addition sequences, in combination with tree-bisection-reconnection (TBR) branch swapping, and with the options MULPARS in effect and STEEPEST DESCENT off [36]. Gaps and random addition replicates were considered as missing bases. A strict consensus tree was generated by setting maxtrees at 20000. The NJ tree was constructed using the program Mega 6.0 with 1000 bootstrap replicates based on the a Kimura-2 parameter distance matrix [37]. Nodes with bootstrap values less than 50 were collapsed.

## Results

### ITS

**Fig 1:**
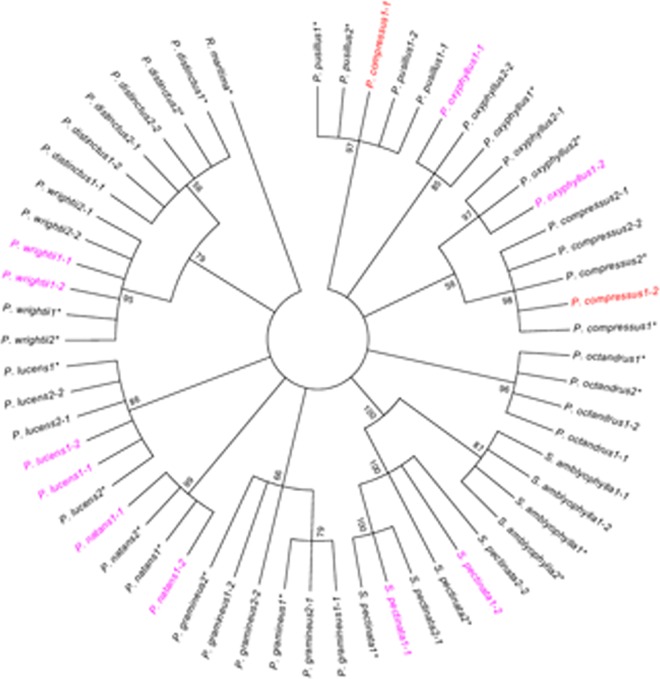
Strict consensus tree for maximum-parsimony (MP) analysis of ITS. Nodes with bootstrap values less than 50 were collapsed. The species marked with asterisk indicated sequences from GenBank. Numbers after the species indicated sampled numbers or accession numbers of the species. Numbers after the horizontal lines indicated the clone number of this sample. The materials colored red indicated congruence between the ITS and *PHYB* phylogenies. The materials colored magenta indicated incongruence between the ITS and *PHYB* phylogenies.

The boundary of ITS was identified by compared with previous published sequences [4, 11, 17]. Length of the ITS sequences for the eight *Potamogeton* species was identical with 630 bp. Length of the two accessions for *Stuckenia amblyophylla* was 654 bp and that for *Stuckenia pectinata* was 645 bp. In addition, existing DNA sequences for twelve *Potamogeton* species were downloaded from GenBank to further evaluate the discriminatory power (S1 Table). Two unique sequences for each material were obtained from five clones. Topology characters of the MP and NJ trees were similar with minor variations in bootstrap values. The MP analysis was set as the heuristic search and limited to 20000 trees. Thus, a strict consensus tree was obtained with the tree length of 446, consistency index (CI) of 0.922, and retention index (RI) of 0.966 (Fig 1). The phylogenetic tree revealed that the two clones of *P*. *compressus1* were separately clustered together with the species *P*. *compressus* and *P*. *pusillus*, which were incompatible with the morphological identification. Nevertheless, identification of the other species using ITS was consistent with morphological features.

### rbcL

**Fig 2:**
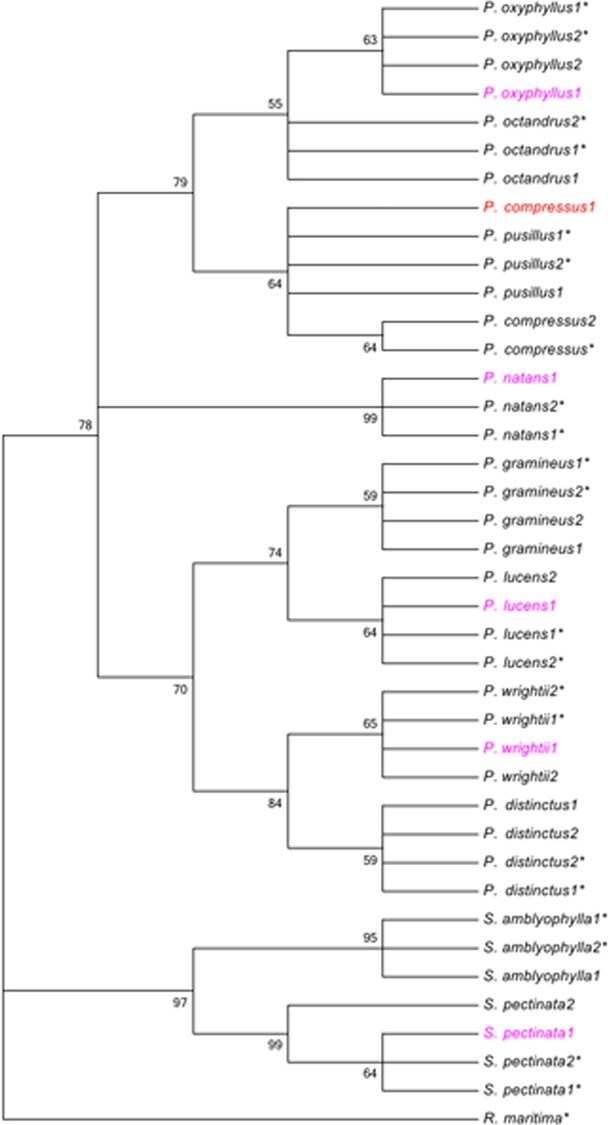
Strict consensus tree for maximum-parsimony (MP) analysis of *rbcL*. Nodes with bootstrap values less than 50 were collapsed. The species marked with asterisk indicated sequences from GenBank. Numbers after the species referred to sampled numbers or accession numbers of the species. The materials colored red indicated congruence between the ITS and *PHYB* phylogenies. The materials colored magenta indicated incongruence between the ITS and *PHYB* phylogenies.

Length of the marker *rbcL* was 881 bp and no sequence variation was observed among multiple sequencings for per sample. For phylogenetic analysis, MP and NJ tree showed similar topology features except minor variations in bootstrap values. The MP analysis yielded a strict consensus tree with the tree length of 91, CI of 0.901, and RI of 0.953. The *rbcL* phylogeny revealed that the maternal parent of *P*. *compressus1* was *P*. *pusillus* (Fig 2). However, identification of the other materials using *rbcL* was consistent with the morphological identification and ITS phylogenetic analysis. For example, the maternal parent of *P*. *lucens1* was *P*. *lucens*; the maternal parent of *P*. *wrightii1* was *P*. *wrightii*; the maternal parent of *P*. *natans1* was *P*. *natans*; the maternal parent of *P*. *oxyphyllus1* was *P*. *oxyphyllus*; and the maternal parent of *S*. *pectinata1* was *S*. *pectinata*.

### PHYB

**Fig 3:**
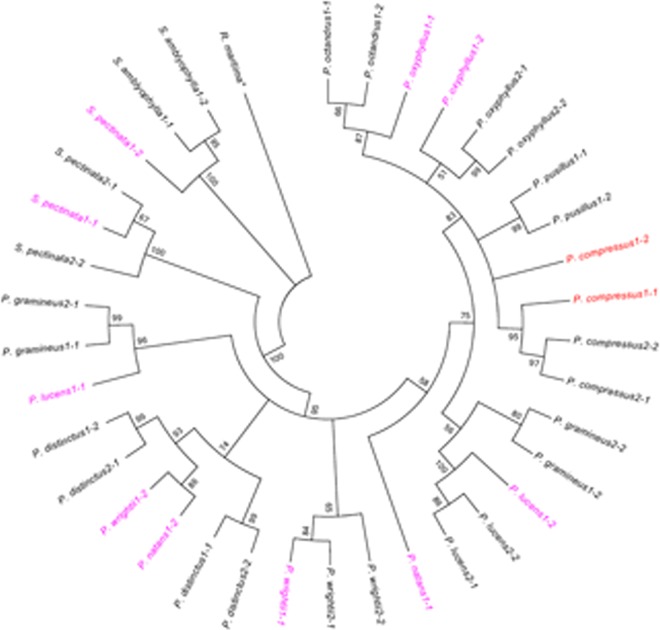
Strict consensus tree for maximum-parsimony (MP) analysis of *PHYB*. Nodes with bootstrap values less than 50 were collapsed. Numbers after the species indicated sampled numbers or accession numbers of the species. Numbers after the horizontal lines indicated the clone number of this species. The materials colored red indicated congruence between the ITS and *PHYB* phylogenies. The materials colored magenta indicated incongruence between the ITS and *PHYB* phylogenies.

Considering the *rbcL* and ITS phylogenies were incompatible, it is necessary to use another marker to elevate resolution and accuracy. In this study, we used a nuclear gene *PHYB* to further improve the discriminatory power. Two accession numbers were obtained for each material and sequence of *R*. *maritime* (AB508058.1) was used as an outgroup in the phylogenetic analysis. Length of *PHYB* was identical with 930 bp. Topology characters of the MP and NJ trees were similar with minor variations in bootstrap values. The MP analysis was set as heuristic search and limited to 20000 trees. The MP analysis yielded a strict consensus tree with the tree length of 447, CI of 0.747, and RI of 0.841. The phylogenetic tree showed that the one clone of *P*. *compressus1* was clustered with *P*. *compressus*, and the other clone probably was clustered with *P*. *pusillus* (Fig 3). Nonetheless, identification of the materials *P*. *oxyphyllus1*, *P*. *lucens1*, *P*. *natans1*, *P*. *wrightii1*, and *S*. *pectinata1* was incongruent with the ITS phylogeny. The *PHYB* phylogeny showed that two clones of *P*. *oxyphyllus1* were separately assembled with *P*. *octandrus* and *P*. *oxyphyllus*; two clones of *P*. *lucens1* were separately assembled with *P*. *gramineus* and *P*. *lucens*; two clones of *P*. *wrightii1* were separately clustered with *P*. *distinctus* and *P*. *wrightii;* and two clones of *S*. *pectinata1* were separately assembled with *S*. *pectinata* and *S*. *amblyophylla*; and one clone of *P*. *natans1* was clustered with *P*. *distinctus*. Nevertheless, two clones of the remaining materials were clustered together, indicating that these materials were pure species, which was consistent with the ITS phylogeny (Fig 3).

## Discussion

*Potamogeton* is a notoriously group in taxonomy due to high morphological and ecological diversity [6, 7]. Moreover, hybridization and polyploidization are prevalent in *Potamogeton* [6, 9]. Traditionally, cytological experiments are used to distinguish the interpretation of hybridization and polyploidization [15]. However, chromosomes for the *Potamogeton* species are extremely small, and it is difficult to use cytological assays accurately to identify them, such as counting chromosome numbers, in-situ hybridization, and karyological characters. In general, molecular barcodes are popular to investigate the phylogenetic relationships in *Potamogeton* [4, 11, 12, 17]. ITS is considered to be one of the most prominent markers in phylogenetic analysis [16]. In this study, the ITS phylogeny demonstrated the material *P*. *compressus1* was a hybrid between *P*. *compressus* and *P*. *pusillus*; and the *rbcL* phylogeny showed that the maternal parent of *P*. *compressus1* was *P*. *pusillus*. The incompatible results contributed to uniparental origin of the cpDNA gene [16, 38].

Low-copy nuclear genes are increasingly applied to obtain a better understanding of phylogenetic analysis, such as *LEAFY* [26, 38], *PHYB* [21, 22], and *CHS* [27]. The low-copy genes are potential to improve the robustness of phylogenetic reconstruction at taxonomy [19]. They are particularly effective to resolve closely interspecific relationships in plants. Moreover, they are helpful to generate strong phylogenetic relationships where universal barcoding markers, such as nrDNA and cpDNA genes, are ambiguous to reveal the internal relationships. *PHYB* is a group of phytochrome family, including *PBYA*, *PHYB*, *PHYC*, *PHYD* and *PHYE* [39, 40]. These proteins act as photoreceptors for red and far-red light in green algae and land plants [41, 42]. Although *PHYB*-related subgroups show an expansion in *Arabidopsis* that *PHYD* and *PHYE* are closely related to *PHYB*, there is no evidence for concerted evolution for *PHYB* [40]. Moreover, *PHYB* has been used for many phylogenetic studies [28, 43]. Southern blot analysis and PCR supply show that *PHYB* is a single-copy gene in grasses except maize [28, 44].

In this study, the first exon of *PHYB* was amplified to identify the *Potamogeton* species. If members of a low-copy gene family are used in the phylogenetic analysis, paralogs should be easily distinguishable. Then we can design specific primers and provide relatively accurate phylogenetic characters [20]. *PHYB* was initially amplified according to the prior research, and then the specific primers were designed in *Potamogeton* [22]. Furthermore, the first exon of *PHYB* is useful to infer the intraspecific relationships with no inferred gaps, unambiguous sequence alignment, and many parsimony informative positions [43, 45].

The *PHYB* phylogeny in this study was the first time to address comprehensively phylogenetic relationships in *Potamogeton*. The ITS phylogeny indicated that *P*. *compressus1* was a hybrid between *P*. *pusillus* and *P*. *compressus*. The maternal parent of *P*. *compressus1* was *P*. *pusillus* based on the *rbcL* phylogeny. Combination analysis of the *PHYB* and *rbcL* phylogenies for *P*. *compressus1* revealed the same conclusion with the ITS phylogeny. These results revealed that species identification solely based on cpDNA genes was controversial and demonstrated that phylogenetic analysis using *PHYB* was receivable [38]. Given limited samples in this study, it is problematic to identify the material *P*. *natans1* solely based on one marker. A combination of *rbcL*, ITS and *PHYB* indicated that *P*. *natans1* was a hybrid between *P*. *distinctus* and *P*. *natans*. Additionally, only one hybrid (*P*. *compressus1*) was identified in the ITS phylogeny. However, the *PHYB* phylogenetic tree revealed that *P*. *oxyphyllus1* was a hybrid between *P*. *octandrus* and *P*. *oxyphyllus*; *P*. *lucens1* confirmed the origin from hybridization between *P*. *gramineus* and *P*. *lucens*; *P*. *wrightii1* was derived from hybridization between *P*. *distinctus* and *P*. *wrightii*; and *S*. *pectinata1* confirmed the origin from hybridization between *S*. *pectinata* and *S*. *amblyophylla*. Previous researches showed that ITS has a high level of homoplasy [16]. ITS homoplasy is prevalent in phylogenetic analysis, owning to compensatory base changes, pseudogene, paralogy, lack of complete concerted evolution, and alignment or sequencing problem [16]. These unpredictable evolutionary events and complex behaviors obviously attenuated its power for phylogenetic analysis. Several researches demonstrated that concerted evolution within different tandem repeats of ITS regions can quickly eliminate one parental pattern and completely homogenized to the other parental lineage [46, 47]. Additionally, ITS probably forms a chimeric mixture of ITS types or maintain biparental types [16, 48]. The incongruence between the ITS and *PHYB* phylogenies revealed that ITS homology was also existed in *Potamogeton*. ITS is homogenized to the maternal lineage for the five hybrids, including *P*. *octandrus* × *P*. *oxyphyllus*, *P*. *gramineus* × *P*. *lucens*, *P*. *distinctus* × *P*. *natans*, *P*. *distinctus* × *P*. *wrightii*, and *S*. *pectinata* × *S*. *amblyophylla*. However, identification of most materials was consistent between the *PHYB* and ITS phylogenies, indicating that the *PHYB* phylogeny was reliable and ITS was helpful for phylogenetic analysis to a certain extent. Thus, a combination of ITS and cpDNA gene is unable to accurately provide evidence for hybrid origin in *Potamogeton*. A three-locus combination of cpDNA, ITS and *PHYB* probably is a potential choice to identify the *Potamogeton* species and further reveal phylogenetic relationships in *Potamogeton*.

In summary, *PHYB* was used to identify the *Potamogeton* species and six hybrids were confirmed for the *PHYB* phylogeny (*P*. *compressus* × *P*. *pusillus*, *P*. *octandrus* × *P*. *oxyphyllus*, *P*. *gramineus* × *P*. *lucens*, *P*. *distinctus* × *P*. *natans*, *P*. *distinctus* × *P*. *wrightii*, and *S*. *pectinata* × *S*. *amblyophylla*).Whereas, only one hybrid (*P*. *compressus × P*. *pusillus*) was identified for the ITS phylogeny. The data indicated that ITS homoplasy was present in *Potamogeton* and ITS was completely homogenized towards one parental lineage. Thus, ITS did not effectively reveal the phylogenetic relationship in *Potamogeton*. A three-locus combination of cpDNA gene, ITS and *PHYB* probably can obtain more robust insights into the evolutionary and phylogenetic relationships in *Potamogeton*.

## Supporting Information

S1 Table Sequences downloaded from GenBank in this study. ID number indicated the names in the phylogenetic trees.
(DOCX)
