# Correction: Identification of Hybrids in *Potamogeton*: Incongruence between Plastid and ITS Regions Solved by a Novel Barcoding Marker *PHYB*

**DOI:** 10.1371/journal.pone.0280043

**Published:** 2022-12-30

**Authors:** Tao Yang, Tian-lei Zhang, You-hao Guo, Xing Liu

After publication of this article [1], the following concerns were raised:

- Some *PHYB* accession numbers reported in Table 1 of this article [1] were also reported in another article (Table S8 of [2]), but the locations and geographical coordinates cited for the same GenBank accession numbers are different in the two articles (see S1 File).
- In [1], *PHYB* GenBank accession numbers KX059488 and KX059485 are reported as associated with two different ID numbers each.

Here, the authors have provided additional information to clarify these issues.

The sequences used in the *PLOS ONE* study [1] were initially deposited in Genbank and the accession numbers reported in Table 1 of [1] accurately reflect the deposit of sequence data obtained from the samples and locations reported in Table 1. The same accession numbers were re-used in [2], where similar sequences were subsequently obtained from different samples at nearby different locations.

The authors have created unique accession numbers for the duplicates of KX059488 and KX059485. The corrected accession numbers and related information are as follows:

GenBank accession number: MW930629

ID: P. gramineus1

Location: Luhuo, Sichuan 100° 13’N, 31° 37’E

GenBank accession number: KX059488

ID: P. gramineus2

Location: Hongyuan, Sichuan 102°30′55.5″, 32°46′43.6″

GenBank accession number: MW930630

ID: P. distinctus1

Location: Tengchong, Yunnan 98° 40’N, 25° 37’E

GenBank accession number: KX059485

ID: P. distinctus2

Location: Chayu, Xizang 97°21′26.6″, 28°37′21″

## Supporting information

S1 File All accession numbers duplicated between the two articles. (XLSX)
