# Supplementary material for: Identification of Hybrids in Potamogeton: Incongruence between Plastid and ITS Regions Solved by a Novel Barcoding Marker PHYB
Source: PLoS One. 2016 Nov 17;11(11):e0166177. doi: 10.1371/journal.pone.0166177 (PMC5113904; doi:10.1371/journal.pone.0166177)
Supplement: S1 Table — ID number indicated the names in the phylogenetic trees. (DOCX) [file pone.0166177.s001.docx]

**Identification of hybrids in** ***Potamogeton*：incongruence between plastid and ITS regions solved by a novel barcoding marker *PHYB***

Tao Yang, Tian-lei Zhang, You-hao Guo*, Xing Liu*

Laboratory of Plant Systematics and Evolutionary Biology, College of Life Science, Wuhan University, Wuhan, Hubei, China.

* Corresponding authors:

You-hao Guo: [yhguo@whu.edu.cn](mailto:yhguo@whu.edu.cn)

Xing Liu: xingliu@whu.edu.cn

**Table S1. Sequences downloaded from GenBank in this study.**

| **Taxon** | **ID number** | **Acession number** | |
| --- | --- | --- | --- |
|  |  | ***rbcL*** | **ITS** |
| *P. wrightii* | *P. wrightii1** | FJ956817.1 | FJ956763 |
|  | *P. wrightii2** | FJ956818.1 | FJ956764 |
| *P. distinctus* | *P. distinctus1** | FJ956814.1 | FJ956759 |
|  | *P. distinctus2** | FJ956815.1 | FJ968813 |
| *P. gramineus* | *P. gramineus1** | FJ956828.1 | DQ840301.1 |
|  | *P. gramineus2** | AB196943.1 | DQ840303.1 |
| *P. lucens* | *P. lucens1** | FJ956829.1 | FJ956774 |
|  | *P. lucens2** | FJ956830.1 | FJ956762 |
| *P. natans* | *P. natans1** | FJ956831.1 | FJ956776 |
|  | *P. natans2** | FJ956832.1 | FJ956777 |
| *P. octandrus* | *P. octandrus1** | FJ956840.1 | JF977907.1 |
|  | *P. octandrus2** | FJ956841.1 | JF977909.1 |
| *P. pusillus* | *P. pusillus1** | FJ956845.1 | FJ151201.1 |
|  | *P. pusillus2** | FJ956846.1 | HQ263485.1 |
| *P. oxyphyllus* | *P. oxyphyllus1** | FJ956853.1 | FJ956798.1 |
|  | *P. oxyphyllus2** | AB196949.1 | DQ840307.1 |
| *S. pectinata* | *S. pectinata 1** | FJ956865.1 | FJ956772 |
|  | *S. pectinata 2** | FJ956866.1 | FJ956770 |
| *S.amblyophylla* | *S.amblyophylla1** | FJ956857.1 | FJ956805.1 |
|  | *S.amblyophylla2** | FJ956855.1 | FJ956803.1 |
| *P. compressus* | *P. compressus1** | AB196846.1 | GU593250.1 |
|  | *P. compressus2** |  | GU593249.1 |
| *R. maritima* | *R. maritima** | HQ901576.1 | JQ034336.1 |

ID number indicated the names in the phylogenetic trees.
